# Common Features of Neural Activity during Singing and Sleep Periods in a Basal Ganglia Nucleus Critical for Vocal Learning in a Juvenile Songbird

**DOI:** 10.1371/journal.pone.0025879

**Published:** 2011-10-03

**Authors:** Shin Yanagihara, Neal A. Hessler

**Affiliations:** RIKEN Brain Science Institute, Wako, Saitama, Japan; Claremont Colleges, United States of America

## Abstract

Reactivations of waking experiences during sleep have been considered fundamental neural processes for memory consolidation. In songbirds, evidence suggests the importance of sleep-related neuronal activity in song system motor pathway nuclei for both juvenile vocal learning and maintenance of adult song. Like those in singing motor nuclei, neurons in the basal ganglia nucleus Area X, part of the basal ganglia-thalamocortical circuit essential for vocal plasticity, exhibit singing-related activity. It is unclear, however, whether Area X neurons show any distinctive spiking activity during sleep similar to that during singing. Here we demonstrate that, during sleep, Area X pallidal neurons exhibit phasic spiking activity, which shares some firing properties with activity during singing. Shorter interspike intervals that almost exclusively occurred during singing in awake periods were also observed during sleep. The level of firing variability was consistently higher during singing and sleep than during awake non-singing states. Moreover, deceleration of firing rate, which is considered to be an important firing property for transmitting signals from Area X to the thalamic nucleus DLM, was observed mainly during sleep as well as during singing. These results suggest that songbird basal ganglia circuitry may be involved in the off-line processing potentially critical for vocal learning during sensorimotor learning phase.

## Introduction

A growing body of evidence suggests the significance of sleep in a variety of learning tasks and processes for memory consolidation [1]. During sleep, patterns of neural activity that occurred during prior waking experience are reactivated in specific brain areas [2]. These behavioral and electrophysiological studies highlight the role of sleep and associated neural activity in the learning of adult animals. Yet, relatively little is known about whether similar neural process might take place in juvenile animals which are engaged in developmental learning, such as vocal practice in infants. Like human speech, birdsong is a complex vocal behavior dependent on learning in early life. Due to the easily quantifiable behavioral output and the discrete neural circuit specialized for learning and producing songs, songbirds have been recognized as an excellent animal for investigating the neural basis of sensorimotor learning in a critical period [3], [4].

**Figure 1:**
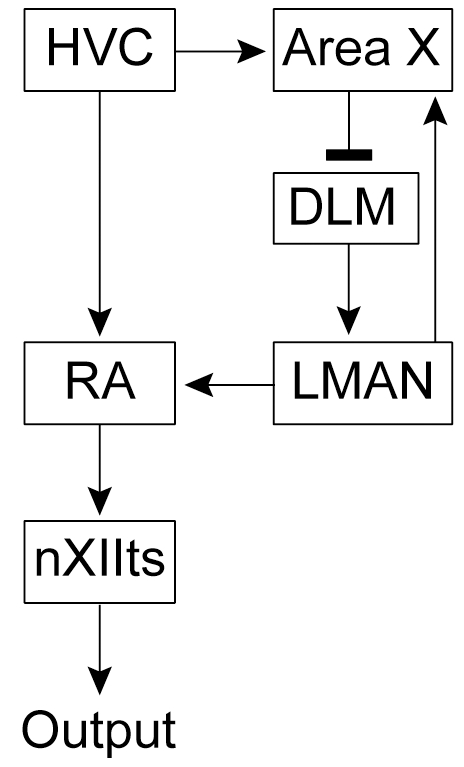
Schematic of the avian song system.

Recent behavioral and electrophysiological studies suggest the importance of sleep in the vocal learning of songbirds. In the course of the vocal learning, juvenile zebra finches exhibit sleep-dependent daily oscillations of singing quality, suggesting that critical neural processes may occur during sleep [5]. Several electrophysiological studies in zebra finches have demonstrated phasic bursts of neural activity in the singing motor circuit during sleep. The motor pathway consists of HVC, the robust nucleus of the arcopallium (RA), and brainstem motor nuclei (**Fig. 1**). This motor circuit is crucial for song production, and neurons in this circuit generate premotor burst activity precisely time-locked to the song output [6], [7]. In sleeping adult birds, RA neurons exhibit spontaneous burst activity which resembles the singing-related premotor burst [8]. Neurons in HVC, which project to RA, also burst concurrent with the RA sleep bursts [7]. In juvenile birds, exposure to tutor song causes a prominent increase in singing-related bursting activity of RA neurons during subsequent sleep [9]. It has been suggested that during sleep, the entire song motor circuit is reactivated in a similar pattern to that which occurs during singing [10].

Another discrete neural circuit crucial for vocal plasticity is the anterior forebrain pathway (AFP, corresponding to mammalian basal ganglia-thalamocortical circuits, **Fig. 1**) [11]. The AFP consists of the basal ganglia nucleus Area X, the medial nucleus of the dorsolateral thalamus (DLM), and the lateral magnocellular nucleus of the anterior nidopallium (LMAN), and is necessary for both juvenile vocal learning and adult song maintenance [12], [13], [14], [15], [16], [17]. Area X contains striatal and pallidal type neurons [18]. Inhibitory pallidal output neurons project to the thalamic nucleus DLM [19]. As in the motor pathway, neurons in Area X are selectively active in awake birds during singing [20], [21], [22], and exhibit selective auditory response to playback of a bird's own song in anesthetized birds [23]. Thus far, it is not known whether neurons in this basal ganglia circuit could show any characteristic spontaneous spiking activity during sleep like that during singing. Since previous studies suggested the importance of sleep in the sensorimotor phase of vocal learning [5], [8], [9], here we focused on juvenile male zebra finches who are engaged in vocal practice. Given the significance of sleep in basal ganglia-dependent vocal learning [5], [8], [9], [10], [24], we aimed to determine whether the activity of Area X neurons of juvenile birds during sleep could share any firing characteristics with those during singing by recording single-unit activity during both states.

## Results

We recorded extracellular single-unit activity from the basal ganglia nucleus Area X of juvenile male zebra finches (24 units, 51–69 days post-hatch). Previous work showed that Area X contains both striatal and pallidal type neurons [18]. Inhibitory pallidal neurons are further subdivided into two types; DLM-projecting and non DLM-projecting [25]. In awake birds, these pallidal neurons can be identified by their high-frequency spontaneous activity during awake non-singing periods, and robust singing-related activity [20], [21]. Neurons with lower spontaneous firing rates during wakefulness (below 30 Hz) are considered striatal type neurons [22]. In this report, based on the high firing rates during awake non-singing states (non-singing firing rate, 110.0±40.9 Hz, mean ± s.d., interquartile range (25th–75th percentile), 91.5–138.1 Hz, 24 units) and singing-related activity describe below, we regard all units as putative pallidal units (hereafter referred to as ‘pallidal’).

**Figure 2:**
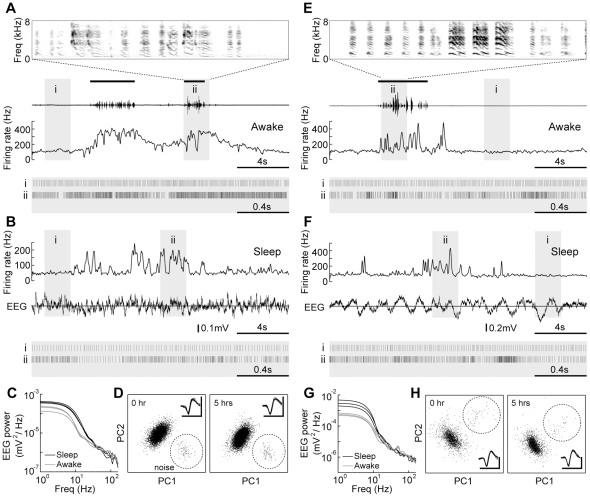
Area X pallidal neurons active during singing were also phasically active during sleep. **A–D,** An example of pallidal unit recorded from a juvenile bird (bird #1, 61 days post-hatch). **A,** Activity during singing. Song (oscillogram, period of singing indicated by black bars) and smoothed firing rate (15 ms gaussian window) are shown. Spectrogram of song (2 second epoch, indicated by gray shade labeled ‘ii’) is shown at the top. Typical spiking activity during non-singing and singing periods (2 second epochs, indicated by gray shades labeled ‘i’ and ‘ii'’, respectively) are shown in raster plots below. **B,** Activity of the same unit during sleep. Simultaneously recorded EEG signal is shown below. Typical spiking activity during tonic and phasic epochs (2 second, indicated by gray shades) are shown by raster plots. **C,** Power spectral densities of 2 second period of EEG during awake (gray, 3 representative traces) and sleep (black, 3 representative traces) periods. Note stronger power of low-frequency band (<10 Hz) during sleep periods. **D,** Unit cluster at initiation of recording and 5 hours later. Black dots are a sorted single-unit. Noise cluster is encompassed with a dotted circle. Insets indicate spike waveforms. Scale bars; 0.3 mV, 1 ms. **E–H,** Another example of pallidal unit (bird #2, 59 days post-hatch). Data are shown as in **A–D**. Despite sleep EEG waveforms appear different in **B** and **F**, power spectral densities for both periods exhibited strong power at low-frequency band, suggesting that those sleep periods can be regarded as slow wave sleep. Audio files (**Audio S1, S2**) are available as **Supporting Information**.

**Figure 3:**
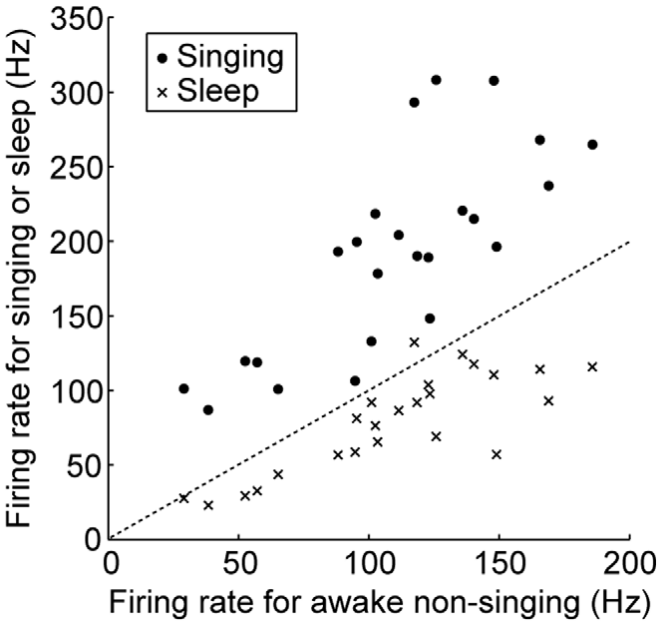
Population summary of firing rates in different behavioral states (24 units). Firing rate during singing (filled circles) was higher than that during awake non-singing state, while the firing rate during sleep (crosses) was lower.

To examine whether similar modulation of activity occurs in Area X during sleep and singing, we evaluated activity recorded from single-units during contiguous awake non-singing, singing, and sleep periods. We found that Area X pallidal units active specifically during singing were also phasically active during sleep (**Fig. 2**). Consistent with previous studies [20], [21], pallidal units fired tonically at high-frequency during awake non-singing periods, and firing rate increased during singing (**Fig. 2A, 2E** **,** **3**, singing firing rate, 191.6±67.1 Hz, mean ± s.d., non-singing firing rate vs. singing firing rate, p = 1.82×10^−5^, 24 units, Wilcoxon signed-rank test). While birds were sleeping in darkness, (**Fig. 2B, 2C and 2F, 2G**, see Materials and Methods), these Area X pallidal units occasionally exhibited phasic increases of activity (**Fig. 2B and 2F**). Since these units fired tonically at a lower rate during most of the sleep period (**Fig. 2B and 2F**), their mean firing rate during sleep (including periods of both phasic and tonic activity) was consistently lower than during awake non-singing periods (**Fig. 3**, sleep firing rate, 79.2±32.8 Hz, awake non-singing vs. sleep, p = 3.43×10^−5^, 24 units, Wilcoxon signed-rank test).

**Figure 4:**
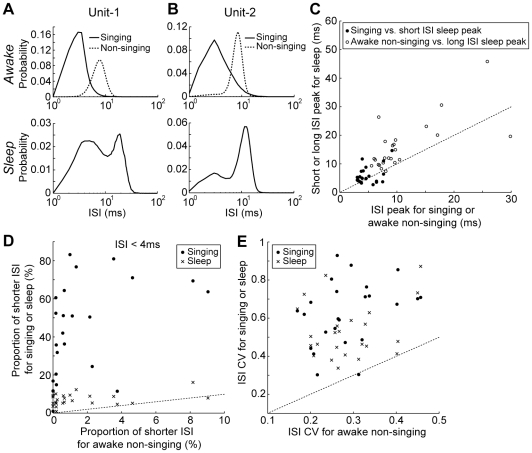
Comparison of interspike interval (ISI) among awake non-singing, singing, and sleep states. **A,** top; ISI distributions of a pallidal unit for awake non-singing and singing period (dotted and solid lines, respectively). bottom; ISI distribution of the same unit for sleep period. Note evident bimodal distribution. The ISI distribution for singing state was generated from 87.1 seconds of singing. The ISI distributions for both awake non-singing and sleep states were generated from 120 seconds of each behavioral state. Data from the same unit appeared in **Fig. 2A–D**. **B,** Another example of ISI distributions for different behavioral states. Data are shown as in **A**. The ISI distribution for singing state was generated from 65.7 seconds of singing, and those for both awake non-singing and sleep states were generated from120 seconds of each state. Data from the same unit appeared in **Fig. 2E–H**. **C,** Comparisons of ISI peak times between awake and sleep state. ISI peak for singing vs. short ISI peaks for sleep (filled circles). ISI peak for awake non-singing vs. long ISI peaks for sleep (open circles). **D,** Population summary of proportion of shorter ISI (ISI <4 ms) for different behavioral states. **E,** Comparison of ISI CV (coefficient of variation) between different behavioral states across population. Data from 24 units in **C–E**.

In order to determine whether similar modulation of Area X activity occurs during singing and sleep periods, we compared several features of the interspike interval (ISI) distribution. For the two representative units shown in **Fig. 2**, the ISI distribution during singing was shifted toward shorter ISIs compared with that during awake non-singing state (**Fig. 4A–B** **,** top). Similar shifts were present for all units (mean ISI; singing, 5.9±2.4 ms, awake non-singing, 11.2±6.9 ms, p = 1.82×10^−5^, 24 units, Wilcoxon signed-rank test). As for the units shown in **Fig. 4A–B**, the ISI distributions of sleep periods tended to overlap with those of awake periods containing both singing and non-singing states. For all units, the ISI distributions during sleep were fit better by bimodal than unimodal Gaussian distributions (R-square values for goodness of fit; bimodal, 0.99±0.01, unimodal, 0.96±0.03, p = 1.82×10^−5^, 24 units, Wilcoxon signed-rank test). The fitted short ISI sleep peak was located similarly to the awake singing ISI peak (singing ISI peak, 4.8±1.7 ms, short ISI sleep peak, 5.9±3.0 ms, p = 0.063, 24 units, Wilcoxon signed-rank test, **Fig. 4C** **,** filled circles), while the long ISI sleep peak was closer to that of awake non-singing state, though shifted to slightly longer ISIs, as described earlier for **Fig. 3** (awake non-singing ISI peak, 10.8±6.2 ms, long ISI sleep peak, 16.1±8.7 ms, p = 2.55×10^−4^, 24 units, Wilcoxon signed-rank test, **Fig. 4C** **,** open circles). These data suggest that the sleep state can include epochs during which activity of Area X pallidal units is similar to that of awake states, both non-singing and singing. In the following, we examine directly whether sleep contains periods in which unit activity dynamics are similar to those during singing.

A prominent feature of Area X pallidal units during singing was phasic burst activity. We first quantified this by calculating the proportion of shorter ISIs (ISI duration <4 ms) for each behavioral state, and confirmed that shorter ISIs were common during singing, but rarely present during awake non-singing periods (**Fig. 4D**, filled circles, proportion of shorter ISI; singing, 43.0±25.0 %, mean ± s.d., awake non-singing, 1.7±2.5 %, p = 1.82×10^−5^, 24 units, Wilcoxon signed-rank test). During sleep, such shorter ISIs were more common than during awake non-singing periods (**Fig. 4D**, crosses, proportion of shorter ISI for sleep, 7.1±3.2 %, sleep vs. awake, p = 3.03×10^−5^, 24 units, Wilcoxon signed-rank test). In sum, the shorter ISIs, which in awake birds exclusively occurred during singing, also frequently occurred during sleep.

In addition to such shorter ISIs, singing often contained periods when Area X pallidal units occasionally paused (i.e. longer ISIs). Such pauses have recently been observed in a subset of Area X pallidal neurons identified as not projecting to the thalamus [21]. We next examined whether such pauses of unit activity occurred during sleep as well as singing. Pauses were defined as ISIs longer than the 99th percentile ISI across all behavioral states, including awake non-singing, singing, and sleep. Across all pallidal units, the mean duration of such longer ISIs was 44.3±31.0 ms (24 units). Half of the single-units (12 / 24) exhibited higher proportion of longer ISIs during awake singing than non-singing states (proportion of longer ISIs; singing, 0.7±0.5 %, awake non-singing, 0.1±0.1 %, mean ± s.d., singing vs. awake non-singing, p = 4.88×10^−4^, 12 units, Wilcoxon signed-rank test). During sleep, the same population of pallidal units also exhibited longer ISIs more frequently (proportion of longer ISIs during sleep, 3.0±1.3 %, sleep vs. awake non-singing, p = 4.88×10^−4^, 12 units, Wilcoxon signed-rank test). In summary, prominent firing features of Area X pallidal units associated with singing, such as shorter or longer ISIs, occurred during sleep, suggesting that neural processing related to singing could take place during sleep as well.

Another common feature of Area X pallidal neurons during singing was a higher level of modulation, including both increases and decreases of firing rate from non-singing level. To examine whether this feature was also present during sleep, we quantified modulation by calculating the coefficient of variation (CV = s.d./mean) of ISIs for each behavioral state. For all units, the variability of ISIs during singing was higher than that during awake non-singing periods (**Fig. 4E**, filled circles, ISI CV during singing, 0.63±0.17, ISI CV during awake non-singing, 0.29±0.08, p = 2.07×10^−5^, 24 units, Wilcoxon signed-rank test). During sleep, the variability of ISIs was also higher than during awake non-singing periods (**Fig. 4E**, crosses, ISI CV during sleep, 0.53±0.14, sleep vs. awake non-singing, p = 1.82×10^−5^, sleep vs. singing, p = 0.049, 24 units, Wilcoxon signed-rank test). Thus, the firing variability of Area X pallidal units during sleep was similar to that during singing.

**Figure 5:**
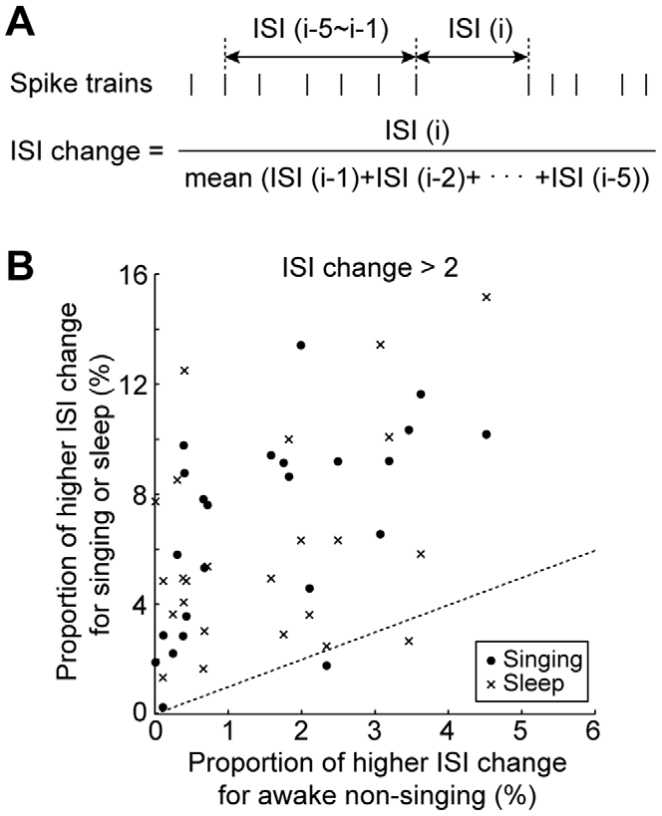
Firing decelerations were frequent during singing and sleep, but rare during awake non-singing periods. **A,** Rapid modulation of firing rate was quantified by calculating ratio of a given ISI to the mean of the preceding 5 ISIs. Vertical ticks indicate schematic spike train. **B,** Comparison of proportion of higher ISI changes (ISI change >2, indicative of firing rate deceleration) between different behavioral states across population (24 units).

Previous in vitro and in vivo electrophysiological studies [26], [27], [28], [29] suggest that decelerations of pallidal firing activity (i.e. a series of shorter ISIs followed by a longer ISI) may be critical for transmitting signals from Area X pallidal output neurons to efferent thalamic neurons in DLM. Thus, we further examined whether the pallidal units could exhibit similar firing decelerations during singing and sleep. To quantify such rapid modulation of activity, we first measured ISI change by comparing a given ISI and a series of the preceding ISIs (**Fig. 5A**, see Materials and Methods). Then, the proportion of the firing rate decelerations, defined as higher ISI change (ISI change >2), was calculated for each behavioral state. Firing rate decelerations occurred frequently during both singing and sleep, but less during awake non-singing periods. Across all pallidal units, the proportion of higher ISI change during singing was greater than that during awake non-singing state (**Fig. 5B**, filled circles, proportion of higher ISI change; singing, 6.8±3.6 %, mean ± s.d., awake non-singing, 1.5±1.3 %, p = 2.35×10^−5^, 24 units, Wilcoxon signed-rank test). Likewise, the proportion of higher ISI change during sleep was greater, and was indistinguishable from that during singing (**Fig. 5B**, crosses, proportion of higher ISI change for sleep, 6.1±3.8 %, awake non-singing vs. sleep, p = 2.35×10^−5^, singing vs. sleep, p = 0.48, 24 units). In summary, Area X pallidal units exhibited decelerations of firing rate predominantly during singing and sleep, suggesting that Area X pallidal output neurons may transmit signals, possibly related to singing, to the downstream thalamic nucleus DLM while a bird was asleep.

**Figure 6:**
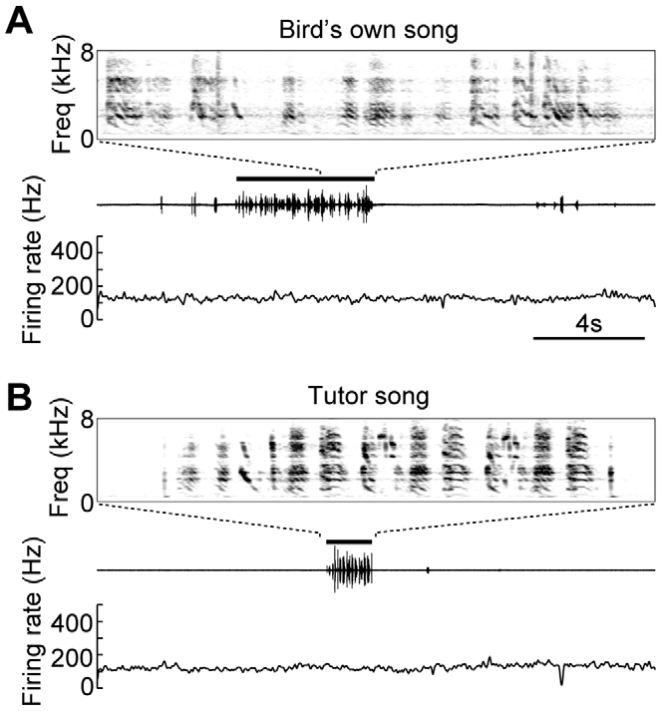
Firing rate of Area X pallidal units was not affected by playback of bird's own song or tutor song during awake period. **A**, An example of pallidal unit activity during playback of bird's own song. Black bar indicates period of song playback. Expanded spectrogram of the bird's own song is shown at the top. **B**, Activity of the same unit during playback of tutor song (black bar), plotted as in panel A. Data from the same unit appeared in **Fig. 2A–D**. Audio files of the bird's own song (**Audio S3**) and tutor song (**Audio S4**) are available as **Supporting Information.**

Previous in vivo electrophysiological studies demonstrated that Area X neurons recorded from anesthetized zebra finches respond selectively to playback of the bird's own song (BOS) [23], [30]. In a subpopulation of our pallidal units (18/24), we further tested for auditory responses to playback of the bird's own song (BOS) or tutor song. In awake birds, the tonic firing of these pallidal units was not altered during playback of BOS or tutor song (Fig. 6, BOS firing rate, 112.6±37.4 Hz, tutor song firing rate, 114.4±38.2 Hz, non-singing firing rate vs. BOS firing rate, p = 0.95, non-singing firing rate vs. tutor song firing rate, p = 0.78, 18 units, Wilcoxon signed-rank test).

## Discussion

Here we report that in juvenile zebra finches, putative pallidal neurons in Area X exhibit phasic spiking activity during sleep which shares some properties with singing-related activity. This suggests a potential off-line role of the basal ganglia network in vocal learning during the sensorimotor learning phase.

Based on various firing properties, the units recorded in this study appear to be of pallidal type. Area X contains striatal and pallidal type neurons [18], and pallidal neurons have been further subdivided into two types; thalamus-projecting and non-thalamus projecting [19], [25]. Since the Area X units we recorded exhibited high spontaneous spiking activity during awake non-singing state and robust singing-related activity, we regarded these units as pallidal neurons [21].

Spontaneous burst spiking activity during sleep has been demonstrated in the song system motor control nuclei. In the motor nucleus RA, singing-related premotor burst activity is reactivated during sleep [8]. The spontaneous burst activity of RA neurons during sleep is driven by RA-projecting HVC neurons which are important for song patterning during singing in adult birds [7]. Furthermore, RA neurons in juvenile birds exhibit increase in sleep burst activity after tutor song exposure in a learned song specific manner [9]. These studies suggested that singing motor nuclei could function in off-line processing related to vocal plasticity of adults and juveniles [10], [24]. Our study showed that pallidal neurons in the basal ganglia nucleus Area X, a part of the anterior forebrain pathway (AFP) critical for juvenile vocal learning, exhibit phasic spiking activity during sleep similar to that present during singing. In juvenile birds, such spontaneous phasic activity of Area X pallidal neurons could influence the RA sleep burst activity by modulating activity of the AFP output nucleus LMAN, although in adults inactivation of LMAN does not affect the RA sleep burst activity [31]. Further study will be necessary to test this possibility, for example by disrupting sleep-related phasic spiking activity in Area X and examining effects on the sleep burst activity in RA. In this report, we described spontaneous phasic activity of Area X pallidal neurons in juvenile birds. As in other song motor nuclei, Area X neurons in adult birds exhibit singing-related activity [20], and similar phasic spiking activity could also be observed while adult birds are sleeping (Yanagihara and Hessler, Society for Neuroscience 2009). This suggests a potential role of Area X sleep-related phasic activity in vocal plasticity of both juvenile and adult birds. Recent studies show that tutoring experience triggers a robust increase in spontaneous sleep-related bursting activity in RA [9] and enhancement of spontaneous synaptic activity in HVC [32]. Thus, it will be important to determine whether the spontaneous phasic spiking activity of Area X neurons could be triggered by experience of tutor song exposure or by singing.

Although several features of spiking activity in Area X pallidal neurons specific to singing also occurred during sleep, patterned reactivation as in song system motor nuclei HVC and RA [7], [8] were not apparent. This may seem to suggest that Area X activity modulation during sleep is not related to that of motor nuclei. Alternately, the modulation during sleep we observed could differ from that of HVC and RA [7], [8] due to the clear distinctions in relationship between activity and behavioral output. In HVC and RA, singing is associated with extremely stereotyped patterns of neural activity [6], [7], [33]. Due to this, the presence of similar stereotyped patterns during sleep periods can be clearly demonstrated. This is not true for Area X pallidal neurons. Activity of these neurons during singing in adult birds is less stereotyped than that in song system motor nuclei such as HVC and RA [20], such that there is weak correlation between song elements and activity patterns. Thus, during singing, a distinct patterned neural activation common to all songs is not present, and would seem difficult to detect during sleep. Moreover, juvenile birds in this study produced ‘plastic songs’ with a high variability of syllable sequence from song to song, and their songs did not form an stereotyped motif structure as in adults (**Figs. S1, S2, S3**). Due to both the lack of clear relationship between song elements and activity even in adults with stereotyped songs, and the high variability of singing behavior in juveniles here, we did not attempt to detect patterned reactivation in this study. Rather, we conclude that sleep modulation we observed is potentially related to previously observed reactivation.

An important question following from our results is the source of sleep-related phasic Area X activity. A potential source to drive Area X phasic activity is excitatory inputs originating from HVC [25]. Area X-projecting HVC neurons exhibit singing-related activity [33], [34], and also show strong burst activity during sleep [31]. Moreover, electrical stimulation of HVC in anesthetized birds evokes excitations and inhibitions in Area X pallidal neurons [29], and the variability of such evoked firing is reduced by dopamine [35]. In this study, we found that shorter and longer ISIs occurred more frequently during singing and sleep than during the awake non-singing state (**Fig. 4D**), and consequently variability of ISIs was higher during singing and sleep (**Fig. 4E**). Thus, HVC may be a potential source to drive such phasic pallidal neurons activity during sleep as well as singing. Since Area X spiny neurons, which are active during singing [22], also receive glutamatergic inputs from HVC [25], they could also exhibit phasic spiking activity during sleep as well. It is possible that phasic activity of Area X pallidal neurons during sleep could also be modulated by inputs from midbrain dopaminergic nuclei such as VTA, potentially related to previously characterized modulation dependent on social context during singing [35], [36], [37], [38], [39], [40], [41].

Acute deceleration of thalamus-projecting pallidal neurons has been considered an important firing property for transmitting signals from Area X to the thalamic nucleus DLM [26], [27], [28], [29]. In the in vitro slice preparation, spiking activity of DLM neurons is driven by stimulation of Area X inhibitory outputs in the absence of excitatory drive [27]. In anesthetized birds, DLM spikes follow deceleration of Area X pallidal neurons activity [26], [28]. In behaving birds, we observed similar decelerations of pallidal neurons activity more frequently during singing and sleep than awake non-singing state (**Fig. 5**), suggesting that Area X pallidal neurons could transmit singing-related signals to DLM during sleep by deceleration of the firing rate.

In contrast to the robust singing-related activity during wakefulness, we did not observe significant auditory responses to playback of bird's own song (BOS) or tutor song in awake juvenile birds. In the in vivo anesthetized adult zebra finches, Area X neurons selectively respond to BOS [23]. The response selectivity of Area X neurons for both BOS and tutor song emerges at around 60 days of age [30], similar to birds used here. This discrepancy in auditory responsiveness between our study and the previous studies could reflect different behavioral states. Indeed, robust auditory responses are found in anesthetized and sleeping birds, but not in awake birds, in some song system nuclei. Such behavioral state-dependent auditory responses are demonstrated in HVC [42], [43], [44], [45], RA [46] and NIf [47]. Though such state-dependent auditory responses are observed in adult birds, HVC multi-unit activity recorded from awake juvenile birds in early sensorimotor phase (<70 days) exhibit tutor song responses [48]. While the issues of state-dependent auditory processing was beyond our scope, further study would be necessary to reconcile this contradiction by recording from both Area X-projecting HVC neuron and Area X neuron simultaneously in juvenile birds and test whether these neurons could be co-modulated by auditory stimuli in a state-dependent manner.

In summary, our results suggest that basal ganglia nucleus Area X may play a role in the off-line processing potentially critical for juvenile vocal learning. In the future study, it is important to determine whether manipulation of such phasic activity during sleep could disrupt normal vocal learning.

## Materials and Methods

### Animals

All experimental procedures and animal care were conducted in accordance with guidelines approved by the RIKEN Animal Experiments Committee (Approval ID: H23-1-223). Electrophysiological data were obtained from fourteen juvenile male zebra finches (range, 51–69 days post-hatch, mean ± s.d., 58±5.0 days). All birds were hatched in our breeding colony, and raised with their parents and siblings until the days of surgery (range, 41–58 days post-hatch, mean ± s.d., 50±5.0 days). During the period of physiological recordings, birds were in the middle portion of sensorimotor learning, as their songs contained some repeats of vocal elements, but with much variability of fine structure across multiple vocalizations, as well as high variability of element patterning (**Fig. S1, S2, S3**, Audio files available as **Supporting Audio files**). Thus, songs had some similarity to those of the tutor, but were clearly distinct (**Fig. 6** **, Fig. S1, S2, S3**).

### Surgical procedures

General procedures for surgery and physiological recordings were described in our previous report [38]. Surgery for implantation of electrodes was conducted under isoflurane anesthesia (1.0–1.3% supplied with air). Each bird was chronically implanted with tetrodes (nichrome wire, diameter, 12.5 µm, impedance, 200–500 kΩ, Kanthal Palm Coast) fixed in a small microdrive, and targeted to Area X (5.1 mm anterior, 1.7 mm lateral from divergence of the central sinus at the border of the forebrain and cerebellum). Single-unit recordings were made from the right (n = 18) or left (n = 6) hemisphere. Since no apparent differences were found, data from both hemispheres were pooled. For electroencephalogram (EEG) recordings, a stainless steel wire (diameter, 200 µm, AM-Systems) was firmly fixed on the brain surface (0.0 mm anterior, 2.0 mm lateral from the central sinus). After surgery, each bird was housed in a recording chamber placed inside a sound attenuation box under a photoperiod of 12 h:12 h LD. Food and water were available ad libitum.

### Electrophysiological recordings

After several days of recovery, single-unit activity was recorded in Area X at a depth of 2.3 – 3.3 mm from the brain surface. All of the units reported here exhibited several firing characteristics consistent with previously described Area X pallidal neurons (such as high frequency spontaneous firing rate, robust singing-related activity, characteristic ISI distributions during singing and non-singing awake state, and spike width), and further singing–related properties were found to be restricted to Area X region of LPO [20]. Thus, we regarded our samples as Area X pallidal units. Single-unit and EEG activity were recorded using Plexon data acquisition system. For single-unit recordings, signals were amplified, filtered (5,000- 20,000-fold, 0.3–9 kHz), and digitized at 40 kHz. The signal-to-noise ratio of isolated single-units was typically greater than 3∶1. For EEG recordings, the signal was amplified, filtered (1,000-fold, 0.7–170 Hz), and digitized at 20 kHz. Once single-unit activity was isolated, recording sessions lasted several hours (7.1±3.0 hours, mean ± s.d.). During recording sessions, we first collected awake non-singing and singing data in illuminated condition (mean duration, 3.9±2.4 hours). Then, lights were extinguished to induce spontaneous sleep (mean duration, 3.2±1.4 hours). Sleep state was determined by visual determination of closed eye, lack of gross body movement, neck muscle relaxation (head-forward or head-backward position), and ongoing EEG slow wave activity. The acoustic signal in the sound attenuation box was simultaneously recorded using a microphone (Model C417, AKG), amplified (DMP3, M-AUDIO) and digitized at 20 kHz. Birds' behavior was monitored using a video camera, and the video data were stored on a hard disk (CinePlex, Plexon). In the song playback experiments, a representative song previously produced by the bird (bird's own song: BOS) or tutor song were repeatedly presented (10 trials for each stimulus) at an intervals of 10 s from a loudspeaker (R10SC, Visaton) placed in a sound attenuation box.

### Data analysis

Analysis was performed using Matlab (Mathworks). The spike signals were manually sorted offline into isolated single-units (Offline Sorter, Plexon). To characterize spike shape for each single-unit, spike width (defined as half width at negative peak deflection) was measured using an averaged spike waveform (average of 10 representative spikes). The spike half width of our samples (mean ± s.d., 0.14±0.02 ms, range, 0.10 – 0.18 ms, n = 24 units) was consistent with that of Area X pallidal neurons extracellulary recorded from juvenile zebra finches [22].

For each single-unit, we calculated mean firing rate, mean interspike interval (ISI), and ISI CV (coefficient of variation, s.d./mean) in each behavioral state (awake non-singing, singing, and sleep). To calculate these values for singing periods, the onset and offset times of songs were manually determined by visual inspection of spectrograms and oscillograms of the acoustic signal (duration of singing period, mean ± s.d., 106.1±61.1 s, n = 24 recording sessions). To obtain these values during awake non-singing and sleep states, we used representative 120-s epochs for each behavioral state. The sleep periods were selected while birds were stably sleeping (1.6±1.1 hours after darkness began). The awake non-singing periods were selected while birds were quiet and stationary. These values for each behavioral state were compared across the population by Wilcoxon signed-rank test.

We also measured percentages of shorter ISI and longer ISI in each behavioral state. The shorter ISI was defined as ISIs less than 4 ms, and the longer ISI was defined as ISIs greater than the 99th percentile of ISI. For each single-unit, threshold of 99th percentile of ISI was set using ISI during all behavioral states (awake non-singing, singing, and sleep). We also quantified rapid changes in firing rate by calculating ‘ISI change’. For a given ISI, the value of ISI change was obtained by calculating the ratio of a given ISI to the mean of the preceding 5 ISIs (**Fig. 5A**). To quantify occurrences of rapid firing rate deceleration, we measured percentages of higher ISI change (defined as ISI change greater than 2) in each behavioral state. The values were compared across the population by Wilcoxon signed-rank test.

Power spectral density (PSD) for EEG signal was calculated using Welch's method for 1-s periods. Consistent with previous EEG measurements in sleeping zebra finches [9], [31], [43], [49], strong EEG power at low-frequency range (less than 10 Hz) was commonly observed during sleep (**Fig.2B–C and 2F–G**). In this study, the EEG data were used to confirm whether birds were sleeping or not. All data are reported as mean ± standard deviation unless noted otherwise.

## Supporting Information

Figure S1 **Examples of juvenile and tutor songs.** **A–C,** Songs recorded from a juvenile bird (56 days post-hatch, bird #3). **D,** Tutor song from an adult bird (bird #4). Spectrogram (top) and oscillogram (bottom) are shown. Audio files are available as **Supporting Information** (Juvenile songs (**A–C**), **Audio S5, S6, S7,** Tutor song (**D**), **Audio S8**).
(TIF)

Figure S2 **Examples of juvenile and tutor songs. A–C,** Songs from a juvenile bird (55 days post-hatch, bird #5). **D,** Tutor song from an adult bird (bird #4). Audio files (Juvenile songs (**A–C**), **Audio S9, S10, S11**, Tutor song (**D**), **Audio S12**).
(TIF)

Figure S3 **Examples of juvenile and tutor songs. A–C,** Songs from a juvenile bird (56 days post-hatch, bird #6). **D,** Tutor song from an adult bird (bird #7). Audio files (Juvenile songs (**A–C**), **Audio S13, S14, S15**, Tutor song (**D**), **Audio S16**).
(TIF)

Audio S1 **Song of bird #1 (61 days post-hatch).** Spectrogram is shown in **Fig. 2A** **.**
(WAV)

Audio S2 **Song of bird #2 (59 days post-hatch).** Spectrogram is shown in **Fig. 2E** **.**
(WAV)

Audio S3 **Song of bird #1 (61 days post-hatch).** Spectrogram is shown in **Fig. 3A** **.**
(WAV)

Audio S4 **Tutor song.** Spectrogram is shown in **Fig. 3B** **.**
(WAV)

Audio S5 **Song of bird #3 (56 days post-hatch).** Spectrogram is shown in **Fig. S1A.**
(WAV)

Audio S6 **Song of bird #3 (56 days post-hatch).** Spectrogram is shown in **Fig. S1B.**
(WAV)

Audio S7 **Song of bird #3 (56 days post-hatch).** Spectrogram is shown in **Fig. S1C.**
(WAV)

Audio S8 **Tutor song (bird #4).** Spectrogram is shown in **Fig. S1D.**
(WAV)

Audio S9 **Song of bird #5 (55 days post-hatch).** Spectrogram is shown in **Fig. S2A.**
(WAV)

Audio S10 **Song of bird #5 (55 days post-hatch).** Spectrogram is shown in **Fig. S2B.**
(WAV)

Audio S11 **Song of bird #5 (55 days post-hatch).** Spectrogram is shown in **Fig. S2C.**
(WAV)

Audio S12 **Tutor song (bird #4).** Spectrogram is shown in **Fig. S2D.**
(WAV)

Audio S13 **Song of bird #6 (56 days post-hatch).** Spectrogram is shown in **Fig. S3A.**
(WAV)

Audio S14 **Song of bird #6 (56 days post-hatch).** Spectrogram is shown in **Fig. S3B.**
(WAV)

Audio S15 **Song of bird #6 (56 days post-hatch).** Spectrogram is shown in **Fig. S3C.**
(WAV)

Audio S16 **Tutor song (bird #7).** Spectrogram is shown in **Fig. S3D.**
(WAV)
