# Supplementary figures and images for: Common Features of Neural Activity during Singing and Sleep Periods in a Basal Ganglia Nucleus Critical for Vocal Learning in a Juvenile Songbird
Source: PLoS One. 2011 Oct 3;6(10):e25879. doi: 10.1371/journal.pone.0025879 (PMC3185046; doi:10.1371/journal.pone.0025879)

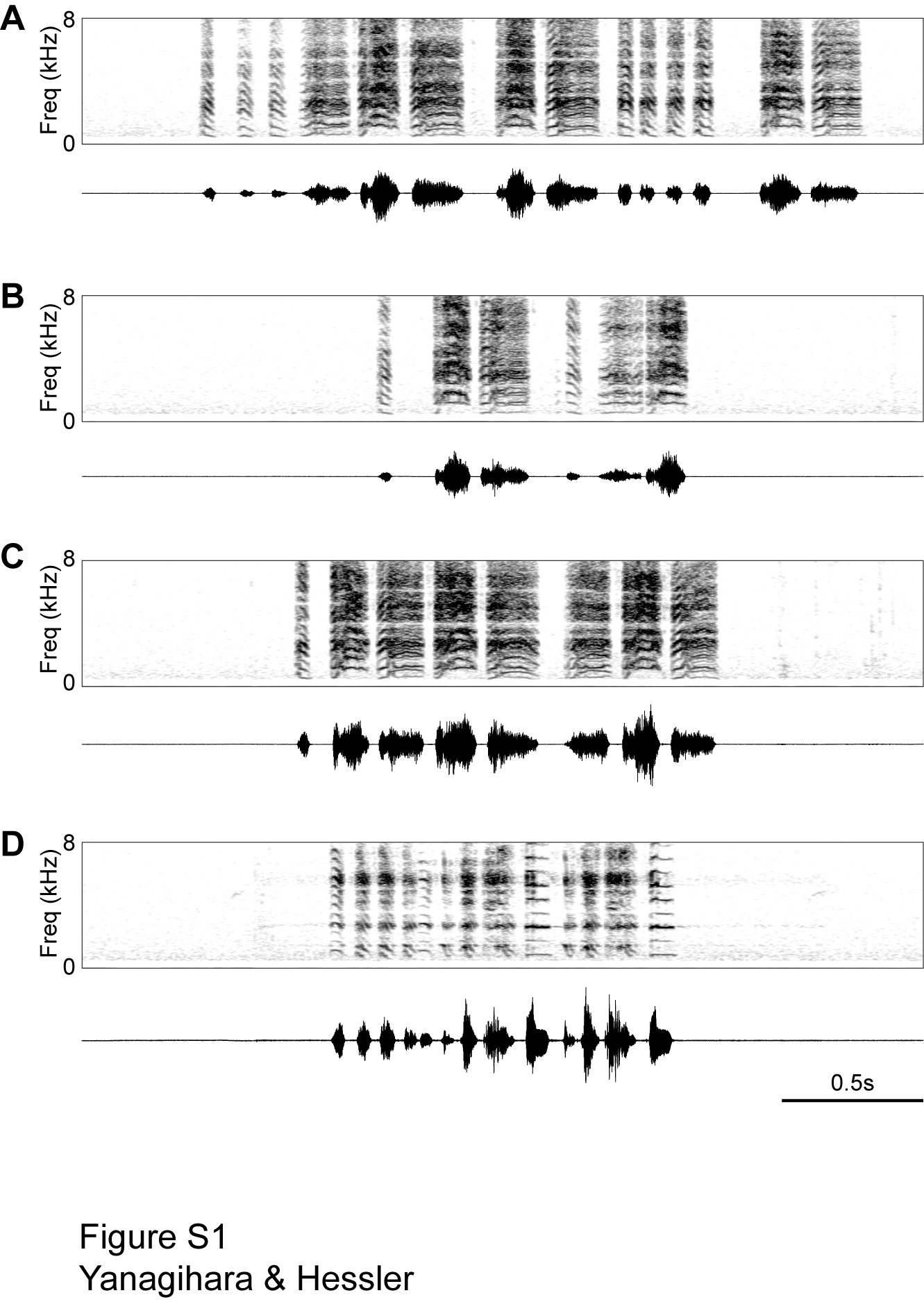

Supplement: Figure S1 — Examples of juvenile and tutor songs. A–C, Songs recorded from a juvenile bird (56 days post-hatch, bird #3). D, Tutor song from an adult bird (bird #4). Spectrogram (top) and oscillogram (bottom) are shown. Audio files are available as Supporting Information (Juvenile songs (A–C), Audio S5, S6, S7, Tutor song (D), Audio S8). (TIF) [file pone.0025879.s001.tif]

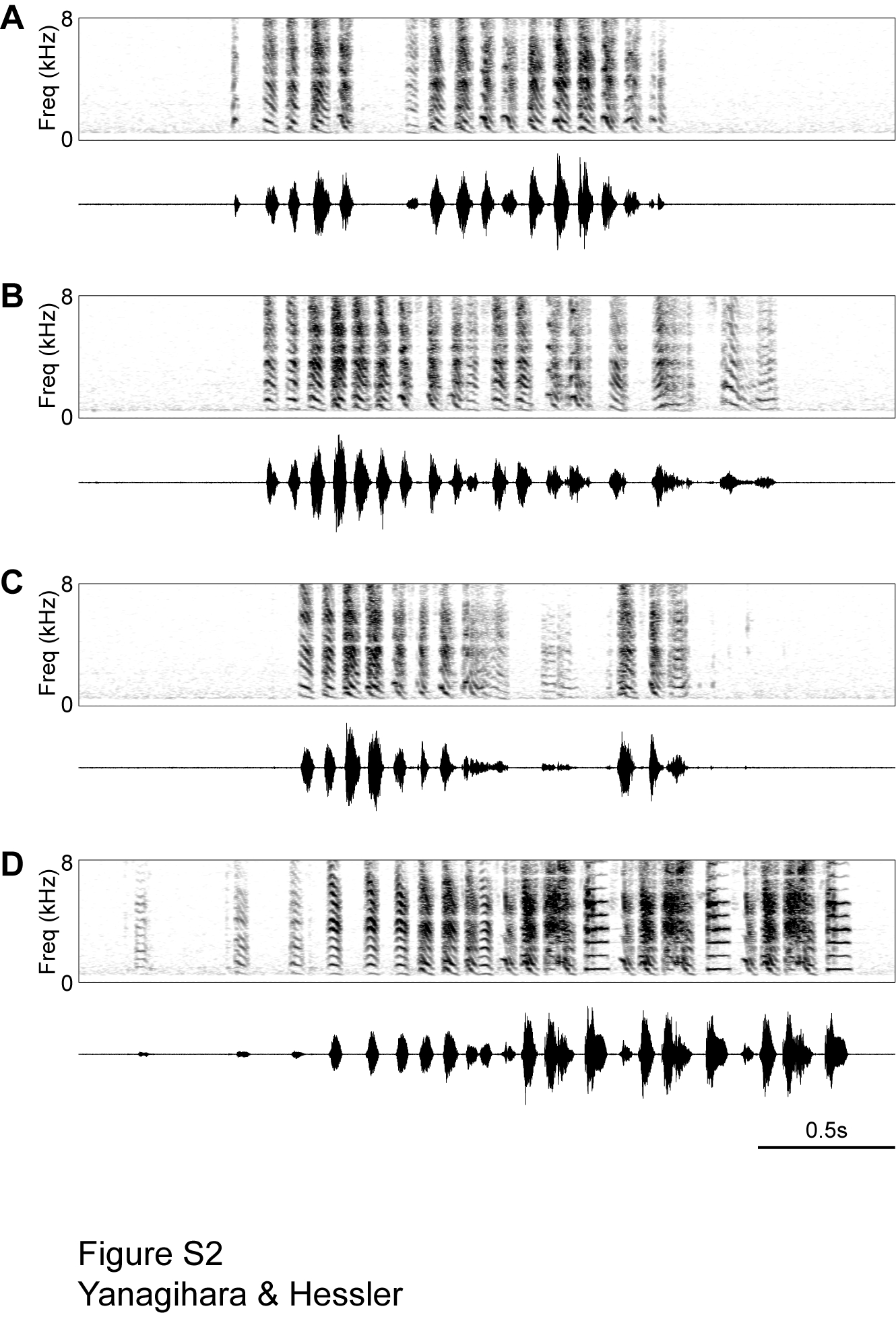

Supplement: Figure S2 — Examples of juvenile and tutor songs. A–C, Songs from a juvenile bird (55 days post-hatch, bird #5). D, Tutor song from an adult bird (bird #4). Audio files (Juvenile songs (A–C), Audio S9, S10, S11, Tutor song (D), Audio S12). (TIF) [file pone.0025879.s002.tif]

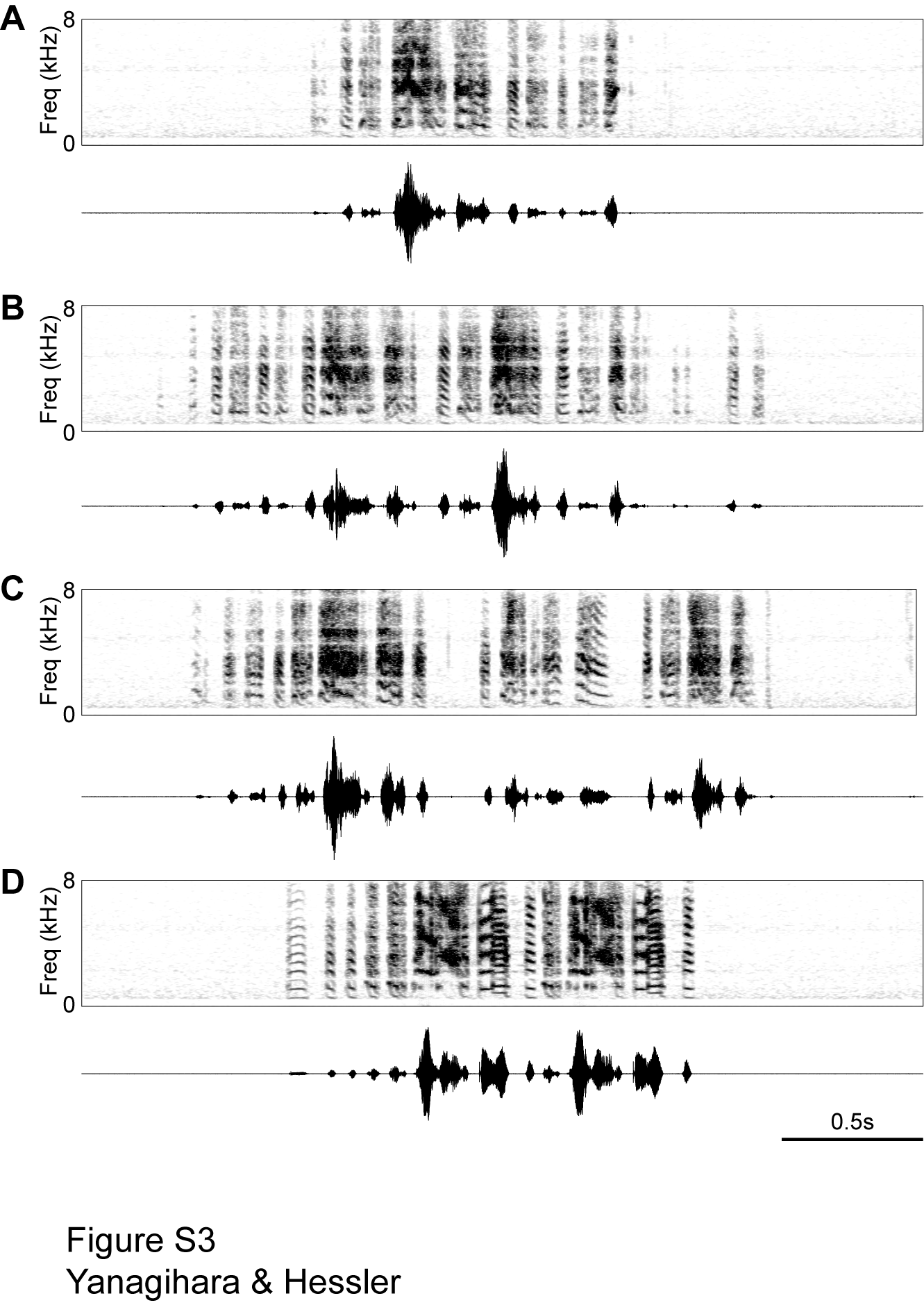

Supplement: Figure S3 — Examples of juvenile and tutor songs. A–C, Songs from a juvenile bird (56 days post-hatch, bird #6). D, Tutor song from an adult bird (bird #7). Audio files (Juvenile songs (A–C), Audio S13, S14, S15, Tutor song (D), Audio S16). (TIF) [file pone.0025879.s003.tif]
